# Exosomes from Bone Marrow Microenvironment-Derived Mesenchymal Stem Cells Affect CML Cells Growth and Promote Drug Resistance to Tyrosine Kinase Inhibitors

**DOI:** 10.1155/2020/8890201

**Published:** 2020-12-13

**Authors:** Xiaoyan Zhang, Yazhi Yang, Yang Yang, Huijun Chen, Huaijun Tu, Jian Li

**Affiliations:** ^1^The Key Laboratory of Hematology of Jiangxi Province, The Department of Hematology, The Second Affiliated Hospital of Nanchang University, 1 Minde Road, Nanchang, 330006 Jiangxi, China; ^2^Basic Medical School, Nanchang University, 465 Bayi Road, Nanchang, 330006 Jiangxi, China; ^3^Graduate School of Medicine, Nanchang University, 465 Bayi Road, Nanchang, 330006 Jiangxi, China; ^4^The Department of Neurology, The Second Affiliated Hospital of Nanchang University, 1 Minde Road, Nanchang, 330006 Jiangxi, China

## Abstract

Although major advances have been achieved in the treatment of chronic myeloid leukemia (CML) by using tyrosine kinase inhibitors, patients relapse after withdrawal and need long-term medication. This reflects the CML clones have not been eliminated completely. The precise mechanisms for the maintenance of CML cells are not yet fully understood. The bone marrow microenvironment constitutes the sanctuary for leukemic cells. Mesenchymal stem cells (MSC) are an important component of the bone marrow microenvironment (BM). It plays an important role in the development and drug resistance of CML. Accumulating evidence indicates that exosomes play a vital role in cell-to-cell communication. We successfully isolated and purified exosomes from human bone marrow microenvironment-derived mesenchymal stem cells (hBMMSC-Exo) by serial centrifugation. In the present study, we investigated the effect of hBMMSC-Exo on the proliferation, apoptosis, and drug resistance of CML cells. The results demonstrated that hBMMSC-Exo had the ability to inhibit the proliferation of CML cells in vitro via miR-15a and arrest cell cycle in the G0/G1 phase. However, the results obtained from BALB/c nu/nu mice studies apparently contradicted the in vitro results. In fact, hBMMSC-Exo increased tumor incidence and promoted tumor growth in vivo. Further study showed the antiapoptotic protein Bcl-2 expression increased, whereas the Caspase3 expression decreased. Moreover, the in vivo study in the xenograft tumor model showed that hBMMSC-Exo promoted the proliferation and decreased the sensitivity of CML cells to tyrosine kinase inhibitors, resulting in drug resistance. These results demonstrated that hBMMSC-Exo supported the maintenance of CML cells and drug resistance in BM by cell-extrinsic protective mechanisms. They also suggested that hBMMSC-Exo might be a potential target to overcome the microenvironment-mediated drug resistance.

## 1. Introduction

Chronic myeloid leukemia (CML) is a disorder caused by the *BCR/ABL* fusion gene. Tyrosine kinase inhibitors (TKI), as one of the most successful molecular targeted drugs in modern medical history, transformed the natural history and prognosis of CML [1]. However, despite the effectiveness and good tolerability of imatinib, drug resistance does emerge [2–4]. Most patients need long-term medication and recur after withdrawal [5]. Some studies have suggested it may be related to the BCR-ABL overexpression, the point mutations in the kinase domain of *BCR/ABL*, *TP53* deletion [6–8], etc. Besides these intrinsic factors, some extrinsic factors are also very important. The hematopoietic microenvironment is considered to be a crucial factor affecting the drug resistance of CML [9]. Mesenchymal stem cells (MSC) are an important component of the bone marrow microenvironment. It can differentiate into a variety of mesodermal cells and secrete multiple bioactive factors to form the tumor microenvironment [10–13]. There is mounting evidence that microenvironmental factors can contribute to CML chemoresistance [14–17]. However, the exact mechanisms of drug resistance remain unclear.

Recently, more and more researchers pay attention to exosomes which can be released by almost all cell types, including the mesenchymal stem cells [18]. Many studies have shown that MSC-derived exosomes (MSC-Exo) have similar functions to the mesenchymal stem cells from which they originate. MSC-Exo may exert various effects on tumor survival, growth, metastasis, and drug response by transferring proteins, mRNA, and microRNA to recipient cells [19–21]. Although the promotion of CML growth and survival induced by bone marrow MSC has been studied, the role of human bone marrow MSC-Exo (hBMMSC-Exo) in these actions remains unclear. In the present study, we investigated the effect of hBMMSC-Exo on the proliferation, apoptosis, and drug resistance of CML cells.

## 2. Materials and Methods

### 2.1. Isolation and Characterization of Bone Marrow MSC

The bone marrow samples were obtained from the Second Affiliated Hospital of Nanchang University, China. Informed consent was obtained from all patients included in the study. MSC were isolated and cultured in DMEM/F12 (Gibco) medium containing 10% fetal bovine serum (FBS, Gibco), 2.0 mM glutamine, penicillin (100 U/mL), and streptomycin (100 U/mL) at 37°C in a humidified atmosphere containing 5% CO_2_.

The immunophenotype of MSC was analyzed by cytofluorimetric analysis. Before experiments, the following monoclonal antibodies were used: anti-CD105-PE, anti-CD34-FITC, anti- CD45-FITC, and anti-CD90-PE (eBioscience, USA). BD FACSDiva flow cytometry (BD FACS Canto™ II, USA) was used. The adipogenic, osteogenic, and chondrogenic differentiation abilities of MSC were determined as previously described.

### 2.2. Chronic Myeloid Leukemia Cells

The primary CML cells (PCML) were harvested from the bone marrow of 16 CML patients. The characteristics of the patients are shown in Table 1. The bone marrow samples were collected by aspiration using a bone marrow needle. All the individuals provided written informed consent. Mononuclear cells were isolated by the Ficoll-Hypaque density-gradient centrifugation, as described previously. 1‐2 × 10^6^ mononuclear cells were seeded in 25 cm^2^ culture flasks (Costar, Corning, Germany) and maintained in RPMI 1640 medium (Gibco) containing 10% fetal bovine serum (FBS, Gibco), 2.0 mM glutamine, streptomycin (100 U/mL), and penicillin (100 U/mL).

The human CML cell line K562 was kindly provided by Prof. Xiaozhong Wang (Nanchang University, Nanchang, China). K562 cells were grown in RPMI 1640 medium (Gibco, CA, USA) supplied with 10% fetal bovine serum (Gibco) and 2 mmol/L L-glutamine (Gibco) at 37°C in a humidified 5% CO_2_ incubator.

### 2.3. Isolation and Characterization of Exosomes

Exosomes released by hBMMSC were isolated and characterized as previously described. For the preparation of exosomes, hBMMSC were cultured in low-glucose DMEM deprived of FBS and supplemented with 0.5% bovine serum albumin (BSA) (Sigma-Aldrich, USA) overnight. Conditioned media were collected every 3 days and centrifugated according to the following steps: centrifugation at 300 g for 10 min to remove the cells in the conditioned media, centrifugation at 2000 g for 20 min to remove the dead cells in the conditioned media, and centrifugation at 10000 g for 30 min to remove the cell debris and protein in the conditioned media. Finally, the supernatant was collected and filtered using 0.22 *μ*m pore filters and centrifuged at 120000 g for 2 h. The supernatant was abandoned, and the isolated pellet was precipitated with 100 *μ*L PBS to obtain the suspension of exosomes.

Exosomes were characterized by using NanoSight LM10 (NanoSight Ltd, Amesbury, UK). The data were assayed using the Nanoparticle Tracking Analysis (NTA) 2.2 analytical software. The expression of CD63, TSG101, and CD81 in MSC-Exo was detected by western blotting. The prepared exosomes were stored at -80°C until further use.

### 2.4. Transmission Electron Microscopy

Purified exosomes were fixed with 4% paraformaldehyde for 2 h. After rinsing, exosomes were ultracentrifuged and suspended in 100 *μ*L PBS. A 20 *μ*L drop of exosomes was loaded onto a Formvar-carbon-coated TEM grid in a dry environment. After exosome adsorption, the grids were washed with PBS and negatively stained with 3% aqueous phosphor-tungstic acid for 5 min. Then, grids were dried and observed by transmission electron microscopy (HITACHI, Japan) at 80 kV.

### 2.5. Cell Proliferation Assay

K562 cells were cocultured with various concentrations of hBMMSC-Exo (0, 50, 100, 150, and 200 *μ*g/mL) for 24, 48, and 72 h. 1 × 10^5^ K562 cells/well were seeded on 96-well plates and incubated in 5% CO_2_ humidified atmosphere at 37°C. Cells were washed twice with PBS and incubated in 100 *μ*L RPMI-1640 (Gibco) containing 10 *μ*L CCK-8 reagent (CCK8 kit, Dojindo, China) for another 3 h. The absorbance of each well at 450 nm was measured using a microplate ELISA reader (model FL 311, Bio-Tek Instruments, USA).

### 2.6. Cell Cycle Analysis

For cell cycle analysis, K562 cells were cocultured with various concentrations of hBMMSC-Exo (0, 50, 100, 150, and 200 *μ*g/mL protein) for 48 h. Cells were fixed with 75% ice-cold ethanol at 4°C overnight. Before staining, K562 cells were washed twice with PBS, added into 100 *μ*L RNase A, and incubated at 37°C for 30 min in the dark. Subsequently, the cells were resuspended in 400 *μ*L propidium iodide (BD Biosciences, USA) at 4°C for 30 min in the dark. The optical density (OD) of each well was detected by flow cytometry (BD FACS Canto™ II, CA, USA) at 488 nm.

### 2.7. Apoptosis Analysis

Apoptosis analysis was performed by using Annexin V/PI staining apoptosis detection kit (BD Biosciences, USA). K562 cells were cocultured with various concentrations of hBMMSC-Exo (0, 50, 100, 150, and 200 *μ*g/mL) for 24 h. Then, imatinib (IM) was added to cultures for a further 24 h. The final concentration of imatinib was 1 *μ*M. The apoptosis of K562 cells was measured according to the manufacturer's recommendations. K562 cells were washed twice with ice-cold PBS then resuspended with Annexin V binding buffer.

The cells were stained with 5 *μ*L FITC-conjugated antiannexin V antibody at room temperature for 20 min and then counterstained with 10 *μ*L propidium iodide (PI). Finally, the cells were analyzed using BD FACSDiva flow cytometry (BD FACS Canto™ II, USA).

### 2.8. Xenograft Tumor Model

To evaluate the in vivo effect of hBMMSC-Exo on K562 cells, female 4-5 weeks old BALB/c-nu mice (Laboratory Animal Center of Academy of Sciences, Hunan, China) were used. All mouse experiments were performed in accordance with the protocol approved by the Animal Care and Use Committee at the Nanchang University. The twenty BALB/c-nu mice were randomly divided into 4 groups (*n* = 5). All groups received subcutaneous injection of 200 mL PBS per mouse into the front of the right backside of the mice containing K562 cells (1 × 10^7^ per mouse) admixed with or without hBMMSC-Exo (200 *μ*g/mL). After a week, the subcutaneous tumor models were successfully established. After two weeks, the treated groups were given imatinib (100 mg/kg), administered by oral gavage daily. Animals were monitored every other day for changes in weight, tumor size, side effects of treatment, and signs of any sickness. The time-point of tumor occurrence was recorded. Tumor growth was evaluated by tumor volume, which was calculated by using the modified ellipsoidal formula: *V* = 1/2 × (length × width^2^). All the mice were sacrificed on day 40 after tumor inoculation, and the subcutaneous tumor was removed en bloc. The tumors were resected, measured, photographed, and made into paraffin slices analyzed by TUNEL and immunofluorescence.

#### 2.8.1. TUNEL Assay

The apoptosis of frozen tissue sections was also measured using a terminal deoxynucleotidyl transferase (TdT-) mediated dUTP digoxigenin nick-end labeling (TUNEL) assay kit (Roche, Switzerland). The protocols were followed according to the manufacturer's instructions. Briefly, tissue sections were dewaxed and rehydrated, washed with 1× PBS, and subjected to DNAse-free proteinase K for 30 min. Next, TACS nuclease reaction mix diluent was added by capillary action and incubated for 20 min. The TUNEL mix was applied to the tissue sections following the manufacturer's instructions and allowed to be incubated at 37°C for 2 h. Before analysis, a DAB assay kit (Boster, China) was used to coloration. Images were taken with an inverted microscope.

#### 2.8.2. Immunofluorescence Staining

Immunofluorescence staining was conducted on frozen tissue sections. Tissue sections were rehydrated through a gradient of alcohol. After washed in PBS for 3 times, tissue sections were blocked in the primary antibody (Caspase3, Proteintech, USA; Bcl-2, Proteintech, USA; GAPDH, Proteintech, USA) for 12-18 h in the fridge at 4°C. The tissue sections were washed for 5 min in 1× PBS for 3 times. The secondary antibody, goat antirabbit IgG (ComWin, China), was diluted at a ratio of 1 : 200 in BGST with buffer solution and incubated at room temperature for 1 h without light. After incubation, the tissue samples were washed for 5 min in 1× PBS for 3 times. 4,6-Diamidino-2-phenylindole (DAPI, Beyotime, China) was added onto the slides before placing a coverslip over the tissue. The coverslips were then sealed and imaged by fluorescence microscopy (Nikon, Japan). The data was analyzed by the Image-pro Plus v6.0 software.

### 2.9. Western Blotting Analysis

The nuclear extracts and total proteins were prepared from the tumor samples of the xenograft tumor model, as described previously. Four groups of tumor samples were milled and lysed in lysis buffer. The protein concentrations were measured with BCA Protein Assay and adjusted to equivalent amounts. Thirty micrograms of protein from each sample was electrophoresed in a 10% SDS-PAGE gel. After electrophoresis, the separated proteins were transferred onto 0.2 mm polyvinylidene fluoride (PVDF) membranes. The membranes were blocked by incubation in 5% nonfat milk in TBS containing 0.1% Tween 20 (TBST) for 1 h at room temperature and then probed with polyclonal antibodies against Bax, Caspase3, Bcl-2, and GAPDH for 1 h. Protein bands were visualized with enhanced ECL detection reagents (ComWin, China).

### 2.10. Statistical Analysis

Statistical analyses were performed with the SPSS 20.0 statistical package for Windows (SPSS, Chicago, USA). The data are expressed as the mean ± standard deviation (SD). Paired data were analyzed using the paired Student's *t*-test. One-way ANOVA was used to evaluate significant differences in cell viability between multiple groups. A *P* value <0.05 was considered statistically significant.

## 3. Results

### 3.1. Characterization of hBMMSC and hBMMSC-Exo

The morphology of MSC from bone marrow was fibroblast-like spindle-shaped (Supplementary Fig. 1A). MSC immunophenotype was checked by cytofluorimetric analysis. More than 95% of the cells lacked hematopoietic-related markers, CD34 and CD45. But more than 96% of the cells were positive for CD90 and CD105 (Supplementary Fig. 1B). The differentiation potential of hBMMSC into adipocytes, osteoblasts, and chondroblasts was confirmed (Supplementary Fig. 1C).

**Figure 1:**
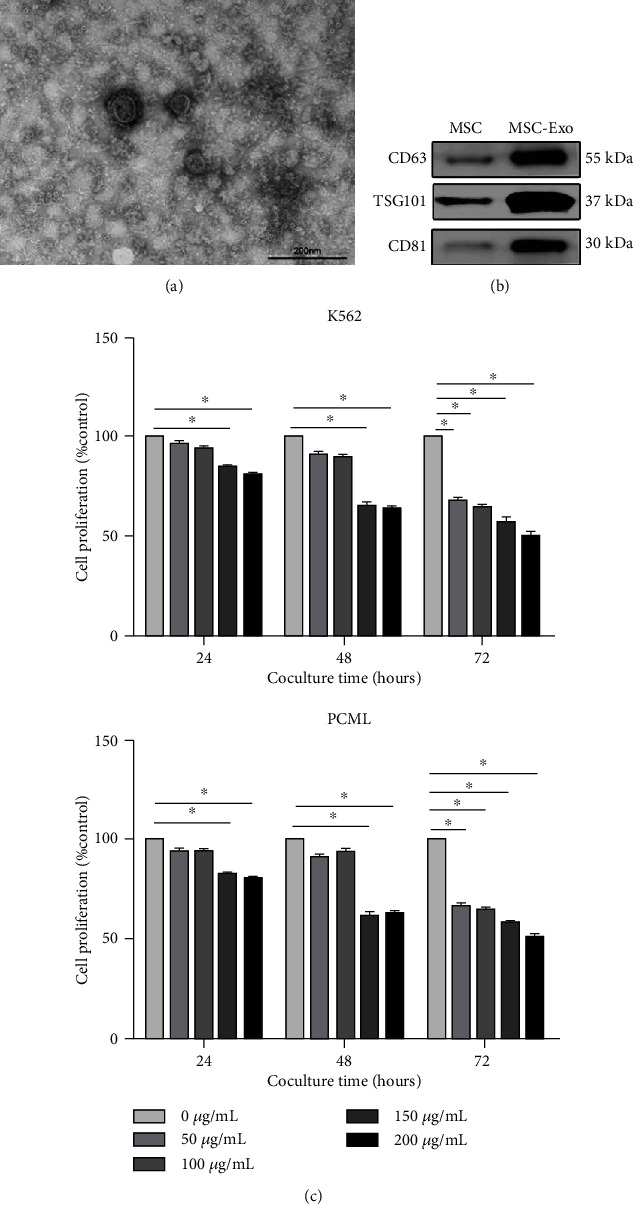
Bone marrow MSC-Exo inhibit CML cell proliferation in vitro. (a) Transmission electron microphotographs of exosomes isolated from bone marrow MSC. The scale bars indicate 100 nm. (b) Detection of CD63, TSG101, and CD81 expression in MSC-Exo by western blotting. (c) Cell proliferation of K562 and PCML cells cocultured with different concentrations of hBMMSC-Exo (0, 50, 100, 150, and 200 *μ*g/mL). The data were expressed as the mean ± SD of the three independent experiments. ^∗^*p* < 0.05 Student's *t*-test.

hBMMSC-Exo were extracted from the hBMMSC culture medium using ultracentrifugation. The morphology and size of exosomes were visualized by TEM (Figure 1(a)). They were heterogeneous, lipid bi-layer, and 100-200 nm in diameter (Supplementary Fig. 2). Western blotting showed that hBMMSC-Exo expressed exosome-specific markers CD81, CD63, and TSG101 (Figure 1(b)). These data demonstrated that hBMMSC-Exo were successfully extracted and purified from hBMMSC culture medium.

### 3.2. hBMMSC Inhibit the Proliferation of CML Cells In Vitro

**Figure 2:**
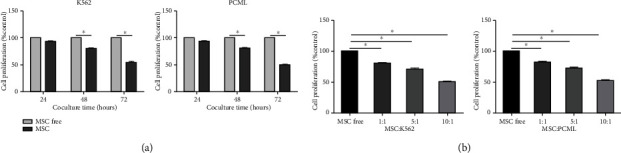
BMMSC inhibit the proliferation of CML cells in vitro. (a) Cell proliferation of K562 cells and PCML cells cocultured with or without different MSC. (b) K562 and PCML cells were cocultured with hBMMSC at different ratios (1 : 1, 5 : 1, and 10 : 1) for 48 h. Then, the proliferation of K562 cells and PCML cells was evaluated. The data were expressed as the mean ± SD of three independent experiments. ^∗^*p* < 0.05 Student's *t*-test.

To determine the effect of hBMMSC on CML cell growth in vitro, we studied the proliferative activity of CML cells after cocultured with hBMMSC. K562 and PCML cells were cocultured with hBMMSC at 1 : 1 ratio for 24, 48, and 72 h. The CCK-8 assay showed that hBMMSC exhibited a time-dependent antiproliferative effect on CML cells (Figure 2(a)), and the inhibitory effect was enhanced over time. After three days of coculture, the cell proliferation of CML cells was inhibited by 49.26% and 45.70%, compared to the control group. Meanwhile, the K562 and PCML cells were cocultured with hBMMSC at different ratios (1 : 1, 5 : 1, and 10 : 1) for 48 h. The result showed that hBMMSC exhibited a concentration-dependent antiproliferative effect on CML cells (Figure 2(b)).

### 3.3. hBMMSC-Exo Inhibit the Proliferation of CML Cells In Vitro via miR-15a

Next, we investigated how hBMMSC inhibit the proliferation of CML cells. The role of exosomes in these actions is not clear. K562 and PCML cells were cocultured with different concentrations of hBMMSC-Exo (0, 50, 100, 150, and 200 *μ*g/mL) and tested for their proliferative activity after 24, 48, and 72 h, as shown in Figure 1(c).

**Figure 3:**
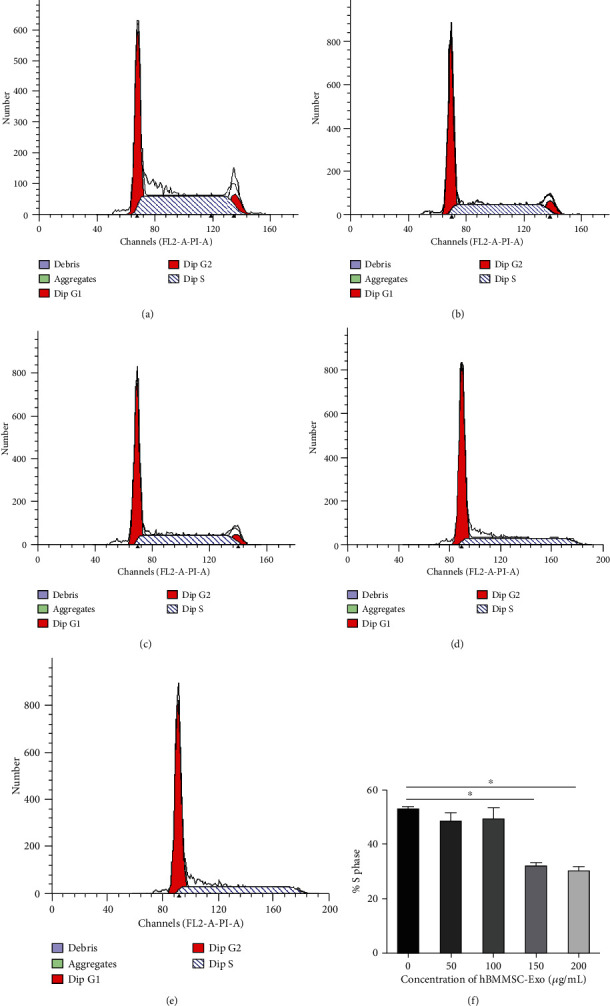
Bone marrow MSC-Exo arrested CML cell cycle. Cell cycle analysis was performed after K562 cells were treated with hBMMSC-Exo (0, 50, 100, 150, and 200 *μ*g/mL) for 48 h: (a) control group; (b) 50 *μ*g/mL group; (c) 100 *μ*g/mL group; (d) 150 *μ*g/mL group; (e) 200 *μ*g/mL group. (f) Representative cell cycle analysis of the S phase in K562 cells after hBMMSC-Exo treatment. The data were expressed as the mean ± SD of the three independent experiments. ^∗^*p* < 0.05 Student's *t*-test.

The CCK-8 assay revealed that hBMMSC-Exo significantly inhibit the proliferation of CML cells in a time- and dose-dependent manner. After the K562 cells were incubated with various concentrations of hBMMSC-Exo, cell cycle analysis was also evaluated (Figure 3). Most of the K562 cells were in quiescence, showing a higher percentage of cells in the G0/G1 phase and a lower percentage of cells in the S phase after coculture with hBMMSC-Exo. This indicated the hBMMSC-Exo arrested the CML cell cycle.

**Figure 4:**
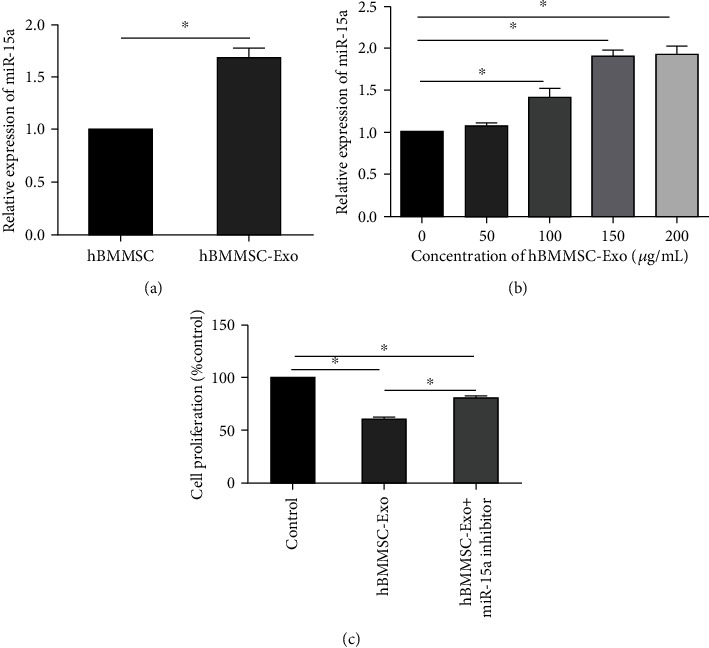
The transfer of miR-15a via hBMMSC-Exo. (a) The relative expression of miR-15a in hBMMSC and hBMMSC-Exo. (b) K562 cells were incubated with various concentrations of hBMMSC-Exo (0, 50, 100, 150, and 200 *μ*g/mL) for 48 h, and the miR-15a levels were evaluated. (c) K562 cells were transfected with miR-15a inhibitor (100 nM) and incubated with hBMMSC-Exo (150 *μ*g/mL) for 48 h. Then, the proliferation of K562 cells was examined. The data were expressed as the mean ± SD of the three independent experiments. ^∗^*p* < 0.05 Student's *t*-test.

MSC-Exo contains a large number of nucleic acids, such as miRNA, mRNA, and mtDNA. miR-15a is the first reported miRNA associated with tumorigenesis. miR-15a can also bind with some cyclin mRNA to regulate cell cycle and inhibit cell proliferation. Thus, we first examined the miR-15a expression levels in hBMMSC and hBMMSC-Exo (Figure 4(a)). The result showed that the levels of miR-15a expression in hBMMSC-Exo were higher than those in hBMMSC. Next, we investigated whether hBMMSC-Exo transferred miR-15a to K562 cells. K562 cells were cocultured with various concentrations of hBMMSC-Exo for 48 h, and then, the levels of miR-15a expression in K562 cells were evaluated (Figure 4(b)). We found that the levels of miR-15a expression in K562 cells increased with the concentrations of cocultured hBMMSC-Exo, suggesting the transfer of miR-15a from hBMMSC-Exo to CML cells. To further confirm that miR-15a shuttled by hBMMSC-Exo might be associated with the proliferation of CML cells, the miR-15a inhibitor was used. After cocultured with miR-15a inhibitor, the proliferation of K562 cells decreased (Figure 4(c)).

### 3.4. hBMMSC-Exo Induce Drug Resistance in CML Cells

**Figure 5:**
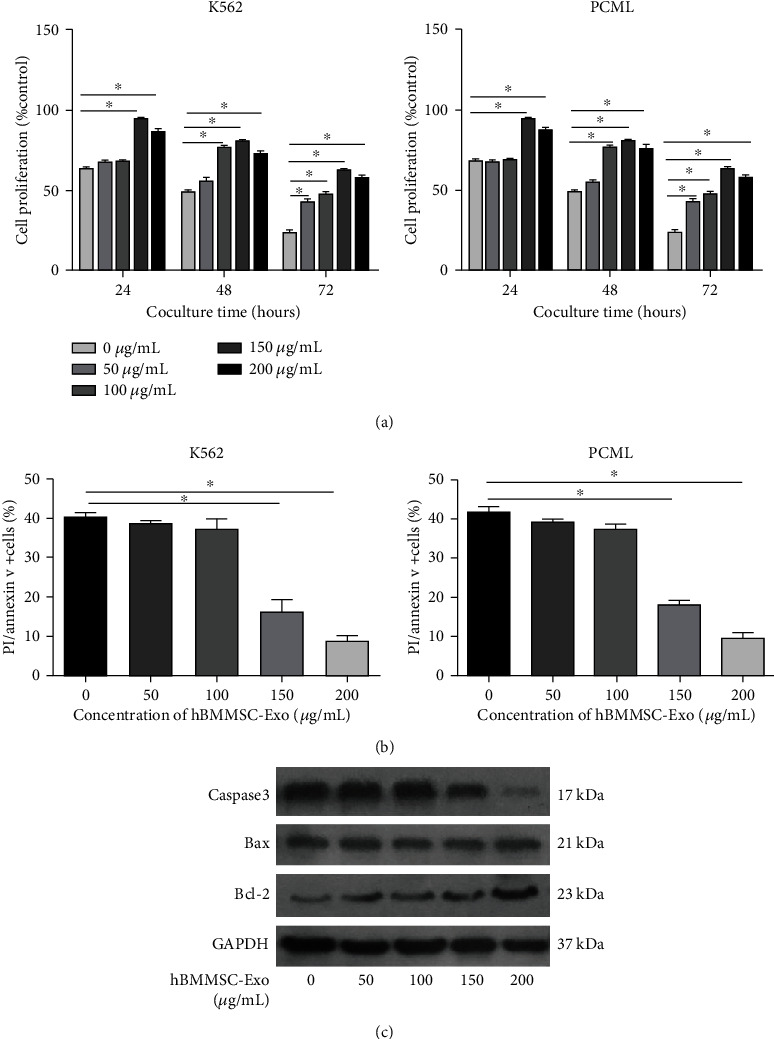
hBMMSC-Exo induce drug resistance in CML cells. (a) The proliferation of K562 and PCML cells after imatinib treatment (1 *μ*M) in the presence of different concentrations of hBMMSC-Exo (0, 50, 100, 150, and 200 *μ*g/mL) for 24, 48, and 72 h. (b) The apoptosis of K562 and PCML cells after imatinib treatment (1 *μ*M) in the presence of different concentrations of MSC-Exo (0, 50, 100, 150, and 200 *μ*g/mL) for 48 h. (c) Representative blots and densitometry analysis of Caspase3, Bax, Bcl-2, and GAPDH in K562 cells after imatinib treatment (1 *μ*M) in the presence of different concentrations of hBMMSC-Exo (0, 50, 100, 150, and 200 *μ*g/mL) for 48 h. The data were expressed as the mean ± SD of three independent experiments. ^∗^*p* < 0.05 Student's *t*-test.

It is known that hBMMSC contribute to the drug resistance of CML cells through cell-to-cell contact or secretion of cytokines. In this study, we examined whether hBMMSC-Exo contributed to this drug resistance. We used IM to examine the drug resistance of CML cells after the hBMMSC-Exo treatment. The results showed coculture with a low concentration of hBMMSC-Exo had no significant effect on the proliferation of CML cells in the presence of IM, but the high concentration of hBMMSC-Exo (150, 200 *μ*g/mL) could significantly increase the proliferation of CML cells (Figure 5(a)).

To study the effect of hBMMSC-Exo on the apoptosis of K562 cells induced by IM, an apoptosis assay was performed by using flow cytometry. hBMMSC-Exo decreased the apoptosis of CML cells (Figure 5(b)) in a dose-dependent manner.

Meanwhile, apoptosis-related proteins Caspase3, Bax, and Bcl-2 were also measured in CML cells after treatment with hBMMSC-Exo in the absence or presence of IM (Figure 5(c)). The antiapoptotic protein Bcl-2 expression increased after the treatment with hBMMSC-Exo, whereas the proapoptotic protein Bax expression did not change. The Caspase3 expression gradually decreased when the CML cells were treated with increased doses of hBMMSC-Exo. These data indicated that hBMMSC-Exo promoted the IM resistance of CML cells by reducing apoptosis and increasing the proliferation of both cells.

### 3.5. hBMMSC-Exo Facilitate Tumor Growth and Induce Drug Resistance In Vivo

**Figure 6:**
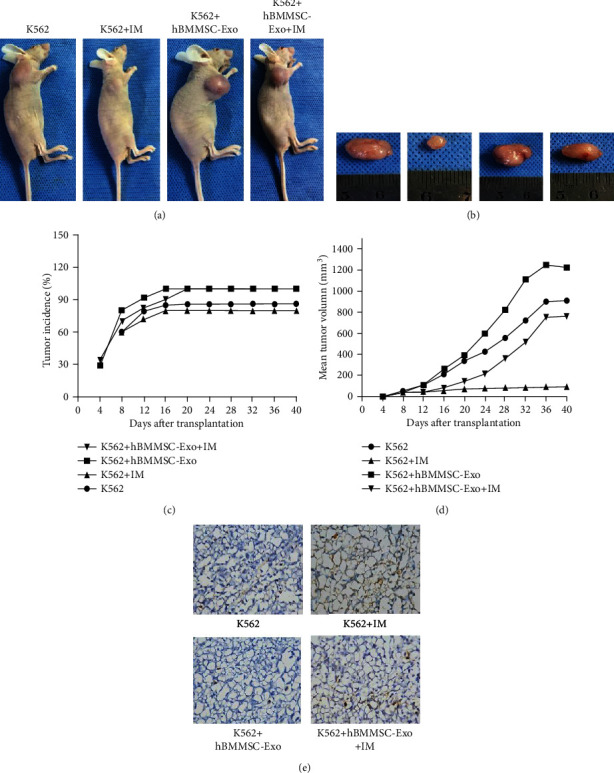
hBMMSC-Exo promote tumor growth in vivo. (a) Representative example of BALB/c-nu mice after transplantation with K562 cells and hBMMSC-Exo by subcutaneous injection. (b) Gross observation of the isolated tumors on day 40 after implantation. (c) Tumor incidence. hBMMSC-Exo significantly increased the tumor incidence. (d) Tumor growth curves. Tumor growth was faster in group K562 cells mixed with hBMMSC-Exo. (e) Representative micrographs of TUNEL staining. The data were expressed as the mean ± SD of the three independent experiments. ^∗^*p* < 0.05 Student's t-test.

To investigate the effect of hBMMSC-Exo on the growth of CML cells in vivo, we transplanted K562 cells and hBMMSC-Exo into BALB/c-nu mice by subcutaneous injection (Figures 6(a) and 6(b)).

In the presence of hBMMSC-Exo, tumor nodules developed in some of BALB/c-nu mice as early as 8 d after inoculation with K562 cells. By 18 d, the tumor incidence in the K562+ hBMMSC-Exo group reached 100% (Figure 6(c)). By contrast, there was no formation of tumor nodules in mice treated with K562 cells alone until 12 d after inoculation. The final tumor incidence was only 70%. Compared to the control group, hBMMSC-Exo significantly increased the tumor incidence.

In addition, tumor growth was faster in the group K562 cells mixed with hBMMSC-Exo than in the group K562 cells alone by measurement of tumor size (Figure 6(d)). The tumor volume of the four groups (K562 alone, K562+hBMMSC-Exo, K562+IM, K562+ hBMMSC-Exo+IM) achieved 278.66 ± 9, 426.31 ± 8, 54.87 ± 3, and 364.09 ± 5 mm^3^, respectively. Meanwhile, after the IM treatment, the tumor nodules of the K562+hBMMSC-Exo group increased rapidly.

**Figure 7:**
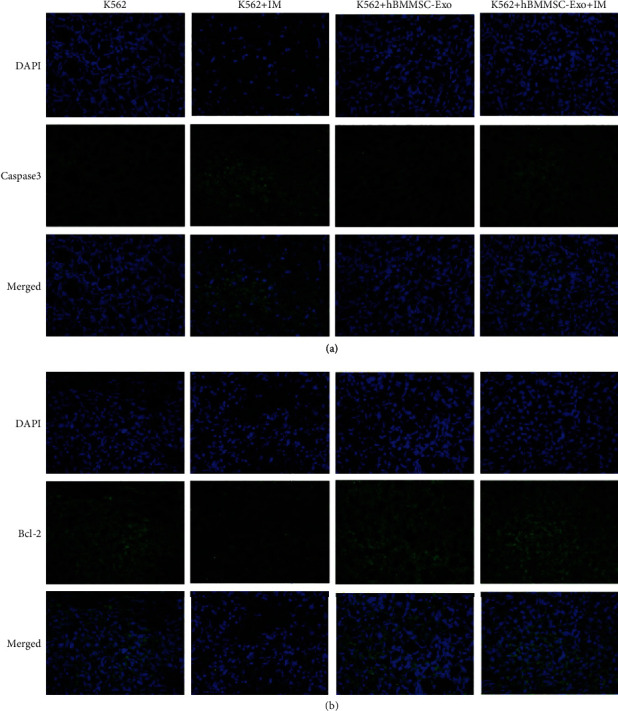
Expression of Caspase3 and Bcl-2 was confirmed by the immunofluorenscence staining in tumor tissues. Nuclei were stained using DAPI. (a) Expression of Caspase3 in tumor tissues by the immunofluorenscence staining. (b) Expression of Bcl-2 in tumor tissue by the immunofluorenscence staining. The data were expressed as the mean ± SD of three independent experiments. ^∗^*p* < 0.05 Student's *t*-test.

TUNEL staining showed the necrotic area of the K562+hBMMSC-Exo+IM group was smaller than that of the K562+IM group (Figure 6(e)). In addition, Caspase3 and Bcl-2 protein of K562 xenograft tissue sections were assessed by immunofluorescence staining (Figure 7). The expression of Caspase3 protein was higher in the K562+hBMMSC-Exo+IM group, while the expression of Bcl-2 protein was lower. The Bax expression was not changed in the four groups.

All these results suggested that hBMMSC-Exo could promote IM resistance of CML cells in vivo.

## 4. Discussion

Although tyrosine kinase inhibitors (imatinib, nilotinib, and dasatinib) are widely used in the treatment of CML patients, these TKI provide only transient antileukemia effects. Patients relapse after withdrawal and need long-term medication. This reflects the CML persistence under TKI therapy [22–26]. The mechanisms of TKI resistance are still poorly understood.

In recent years, accumulating evidence indicates that exosomes play a vital role in cell-to-cell communication. Exosomes can transfer not only membrane components but also functional mRNAs, miRNAs, and proteins, which may reprogram acceptor cells [27, 28]. These studies have reported that exosomes may exert various effects on the growth, metastasis, angiogenesis, and drug response of different tumor cells. It has been demonstrated that MSC-Exo inhibited both in vitro and in vivo growth of HepG2 hepatoma, Kaposi's sarcoma (KS), and Skov-3 ovarian cancer cell lines. The genes related to the antiproliferative pathway were upregulated, such as GTP-binding RAS-like 3 (DIRAS3), retinoblastoma-like 1 (Rbl-1), and cyclin-dependent kinase inhibitor 2B transcript (CDKN2B) [27]. Exosomes derived from human cord blood Wharton's jelly MSCs have also been reported that it can inhibit bladder tumor T24 cell growth and induce apoptosis in vitro and in vivo [29]. In addition, in multiple myeloma (MM), normal hBMMSC-Exo inhibited the growth of MM cells, while MM hBMMSC-Exo promoted MM tumor growth [30, 31]. On the basis of this information, MSC-Exo could therefore exert either antiproliferation or proapoptotic effects on tumor cells. However, the role of MSC-Exo from human bone marrow in CML cell growth, progression, and drug resistance has not yet been thoroughly investigated.

We successfully isolated and purified exosomes from hBMMSC by serial centrifugation. The results demonstrated that hBMMSC-Exo could inhibit the proliferation of CML cells in vitro via miR-15a and arrest cell cycle in the G0/G1 phase. Such inhibition was likely to confer tumor cells a better survival by preserving their proliferative capacity. However, the results obtained from BALB/c nu/nu mice studies apparently contradicted the in vitro results. In fact, hBMMSC-Exo increased tumor incidence and promoted tumor growth in vivo. Further study showed the antiapoptotic protein Bcl-2 expression increased, whereas the Caspase3 expression decreased. Similar to ours, researchers have found that exosomes could increase tumor growth in BALB/c nu/nu mice xenograft model by facilitating angiogenesis and tumor cell proliferation. But exosomes did not directly stimulate the proliferation of these cells; they instead activated angiogenesis by increasing the VEGF expression in tumor cells, which might favor tumor engraftment and growth [29].

Our results also showed hBMMSC-Exo could decrease the sensitivity of CML cells to tyrosine kinase inhibitors and promote IM resistance of CML cells by reducing apoptosis and increasing the proliferation of both cells in vivo, while one recent study indicated that exosomes derived from human umbilical cord mesenchymal stromal cells (hUCMSC-Exo) promoted IM-induced cell viability inhibition and apoptosis by enhancing the increased Bax expression and the decreased Bcl-2 expression and activating Caspase9 and Caspase3 [32]. This is different from our experimental results. As the author analyzed, the apoptosis was affected by many factors, the most important of which was the sensitivity of K562 cells to IM. Although K562 cells are one cell line, they are not absolutely homogeneous. The apoptosis rate of different experiments could not be compared. Therefore, it was difficult to analyze apoptosis rates between different studies. In addition, because the exosomes came from different MSC, they might contain different substances.

It has been reported that hBMMSC-Exo can induce resistance of MM cells to bortezomib (BTZ) [33, 34]. hBMMSC-Exo could inhibit the reduction of the Bcl-2 expression caused by BTZ and reduce the cleavage of Caspase9, Caspase3, and PARP. Researchers also found hBMMSC-Exo could decrease the sensitivity of BM2 cells to docetaxel. In addition, the exosomes from rat bone marrow-derived MSC (rBMMSC-Exo) can protect the rat pheochromocytoma PC12 cell glutamate-induced excitotoxicity. In this study, it was also revealed that rBMMSC-Exo increased Akt phosphorylation and Bcl-2 expression and reduced the expression of Bax [35].

Our present study provided novel evidence of the involvement of hBMMSC-Exo in CML cell growth and drug resistance. They also suggested that hBMMSC-Exo might be a potential target to overcome microenvironment-mediated drug resistance.

## 5. Conclusion

In summary, the BM microenvironment provides an extremely intricate sanctuary for CML cells. Here, our results highlighted a novel communication mechanism between hBMMSC and CML cells through exosomes and demonstrated that hBMMSC-Exo could inhibit the proliferation, decrease the sensitivity of CML cells to tyrosine kinase inhibitors, and promote IM resistance of the CML cells.

## Figures and Tables

**Table 1:** The characteristics of the 16 patients with CML.

Patient number	Disease status	Gender	Age	WBC count (×109/L)	Karyotype	Anti-CML therapy
1	Chronic phase	M	60	138.5	t(9;22)(q34;q11)	None
2	Chronic phase	M	54	75.6	Complex	None
3	Accelerated phase	F	49	105.2	t(9;22)(q34;q11)	None
4	Chronic phase	F	51	46.3	t(9;22)(q34;q11)	None
5	Chronic phase	M	53	237.9	t(9;22)(q34;q11)	None
6	Accelerated phase	F	64	392.3	t(9;22)(q34;q11)	None
7	Accelerated phase	F	47	153.4	t(9;22)(q34;q11)	None
8	Blastic crisis	F	55	242.7	t(9;22)(q34;q11)	None
9	Blastic crisis	M	58	175.8	Complex	None
10	Chronic phase	M	39	409.1	t(9;22)(q34;q11)	None
11	Chronic phase	M	47	364.5	t(9;22)(q34;q11)	None
12	Chronic phase	F	62	283.4	t(9;22)(q34;q11)	None
13	Accelerated phase	M	60	196.7	t(9;22)(q34;q11)	None
14	Accelerated phase	F	44	83.5	t(9;22)(q34;q11)	None
15	Chronic phase	M	45	252.4	Complex	None
16	Chronic phase	M	47	375.9	t(9;22)(q34;q11)	None

## Data Availability

The original data used to support the findings of this study are available from the corresponding author upon request.

## Supplementary Materials

Supplementary Materials Supplementary Figure 1: identification of hBMMSC. Supplementary Figure 2: particle size distribution in purified hBMMSC-Exo.
