# Supplementary material for: Exosomes from Bone Marrow Microenvironment-Derived Mesenchymal Stem Cells Affect CML Cells Growth and Promote Drug Resistance to Tyrosine Kinase Inhibitors
Source: Stem Cells Int. 2020 Dec 13;2020:8890201. doi: 10.1155/2020/8890201 (PMC7752271; doi:10.1155/2020/8890201)
Supplement: Supplementary Materials — Supplementary Figure 1: identification of hBMMSC. Supplementary Figure 2: particle size distribution in purified hBMMSC-Exo. [file 8890201.f1.pdf]

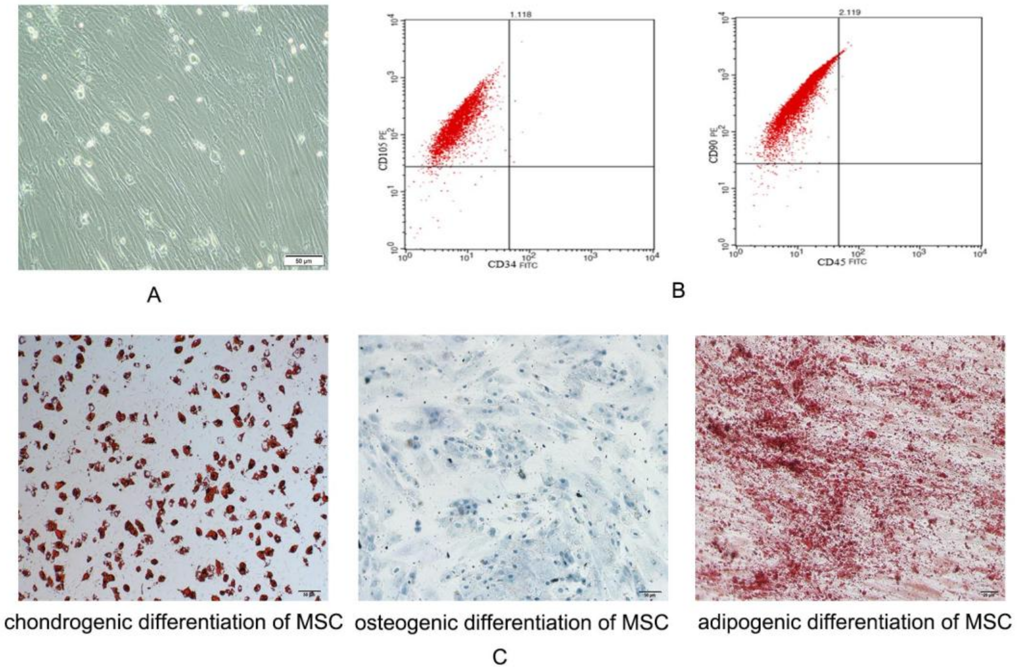

Supplementary Figure 1. identification of hBMMSC.

(A) Representative morphology of confluent MSC layers from bone marrow. (B) The immunophenotype of primary MSC. (C) The multilineage differentiation ability of MSC (chondrogenic, osteogenic, and adipogenic differentiation).

Supplementary Figure 2. Particle size distribution in purified hBMMSC-Exo.

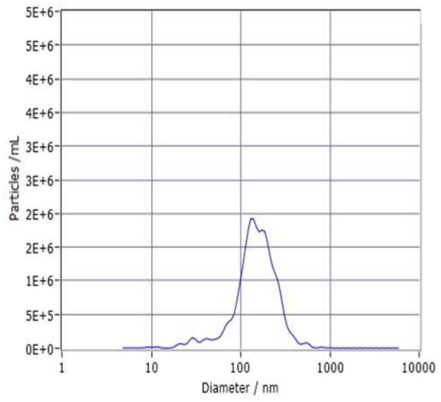

Peak Analysis (Concentration)

Diameter / nm	Particles/mL	FWHM / nm	Percentage
142.4	1.9E+6	166.8	96.5
29.1	1.5E+5	10.2	2.9
11.3	1.5E+4	4.2	0.3
800.3	1.4E+4	114.7	0.1
1208.8	3.2E+3	163.4	0.0
